# A Human Support Robot for the Cleaning and Maintenance of Door Handles Using a Deep-Learning Framework

**DOI:** 10.3390/s20123543

**Published:** 2020-06-23

**Authors:** Balakrishnan Ramalingam, Jia Yin, Mohan Rajesh Elara, Yokhesh Krishnasamy Tamilselvam, Madan Mohan Rayguru, M. A. Viraj J. Muthugala, Braulio Félix Gómez

**Affiliations:** 1 Engineering Product Development Pillar, Singapore University of Technology and Design (SUTD), Singapore 487372, Singapore; rajeshelara@sutd.edu.sg (M.R.E.); madan_rayguru@sutd.edu.sg (M.M.R.); viraj_jagathpriya@sutd.edu.sg (M.A.V.J.M.); brauliofenixgomez@hotmail.com (B.F.G.); 2 Department of Electrical Engineering, University of Western Ontario, London, ON N6A 3K7, Canada; ykrishn4@uwo.ca

**Keywords:** HSR, door handle cleaning, human service robot, cleaning, object detection, deep learning

## Abstract

The role of mobile robots for cleaning and sanitation purposes is increasing worldwide. Disinfection and hygiene are two integral parts of any safe indoor environment, and these factors become more critical in COVID-19-like pandemic situations. Door handles are highly sensitive contact points that are prone to be contamination. Automation of the door-handle cleaning task is not only important for ensuring safety, but also to improve efficiency. This work proposes an AI-enabled framework for automating cleaning tasks through a Human Support Robot (HSR). The overall cleaning process involves mobile base motion, door-handle detection, and control of the HSR manipulator for the completion of the cleaning tasks. The detection part exploits a deep-learning technique to classify the image space, and provides a set of coordinates for the robot. The cooperative control between the spraying and wiping is developed in the Robotic Operating System. The control module uses the information obtained from the detection module to generate a task/operational space for the robot, along with evaluating the desired position to actuate the manipulators. The complete strategy is validated through numerical simulations, and experiments on a Toyota HSR platform.

## 1. Introduction

Cleaning and disinfecting are very important steps in preventing the acquisition and spread of infectious diseases inside closed environments such as apartments, community centers, shopping malls, hospitals, etc. In particular, high-contact-point areas such as doors, lifts, handrails, etc. are major sources of contamination. In [1], the authors reveal that the door handle and its connected areas are highly sensitive contact points that are prone to be contaminated, and a key medium for spreading the germs. Therefore, the frequent cleaning or disinfection of doors handles is essential for preventing the acquisition and spread of infection. However, due to a shortage of manpower, the frequent cleaning of indoor areas has become a key challenge [2]. In addition to that, involving manpower carries a high risk of infection while working for long time in such areas. Mobile robots can be used as a viable solution to the problems associated with conventional cleaning and disinfecting methods due to their proven ability to assist humans in diverse application areas such as hospitals, elderly homes, and industries.

Many robotic solutions have been designed and developed, targeting routine tasks in healthcare facilitie. The works [3,4] demonstrate that service robots are used to deliver food and medicine. UV-light-installed mobile robot was introduced by Danish-based company UVD Robot [5] for efficiently disinfecting hospital rooms, which could slow the disease spread through viruses. These robots can disinfect anything much better than other techniques, using a mobile array of powerful short-wavelength ultraviolet-C (UVC) lights that emit enough energy to shred the DNA or RNA of any microorganisms exposed to them. In [6], author proposed an autonomous robot to performs bed baths in the pursuit of patient hygiene. In addition to this core functionality, the robot is equipped with a fall-detection system consisting of a video camera and a 3D LiDAR to identify patients who have fallen to the ground. The robot can notify the medical staff for the assistance in an emergency condition of a person detected by the fall detection module and remote condition monitoring module. GeckoH13 [7] has been developed as an automated solution for the cleaning of walls in health sectors.This robot can climb on walls with the aid of vacuum adhesion. The task is carried out by spraying different liquid mixers apart from steam. GeckoH13 is effective in sterilizing uniform wall surfaces to a great extent. In [8,9,10,11,12,13,14] mobile robot platform had demonstrated for indoor and outdoor cleaning application such as floor cleaning [8,9], lawn mowing [10], wall cleaning [15], window cleaning [16], pool cleaning [12], pavement [11] and external facade cleaning [14]. Similarly, the service robots are utilized for cleaning the table in food courts [13] and collecting the used plates from the customer.

However, the cleaning of sensitive contact points such as door handles is highly challenging for service robots. To carry out this task, the service robot needs an optimal vision system to identify the target object and the surrounding environment, and generate a trajectory planning scheme corresponding to arm-manipulation function to safely perform the proper cleaning tasks. In the literature, various techniques have been developed for mobile robots to recognize a door and door handle, such as laser scanner technique, ultrasonic sensors, and image-processing schemes. Here, laser scanning and ultrasonic sensors schemes have lot of limitations. They work mostly on a known model of a door and accuracy is not good [17]. Image-processing-based schemes are widely studied for mobile robot object-recognition tasks. These are also widely studied for door and door-handle detection tasks. A door-frame detection model was proposed using Hough Transform to detect edges, and a fuzzy logic-based algorithm was used to find the relationship between them [18]. However, the model was unable to differentiate doors from large objects typically indoors. In another work, a computer vision-based door-detection algorithm was reported for blind people conducting indoor activity [19]. Here, the author used the generic geometric of a door model and the edges and corner information for detecting doors. In another study [20], Huy-Hieu Pham used Kinect sensor-generated point cloud data and a 3D-image-processing scheme to detect a door and other indoor objects. In this work, the author uses a Voxel Grid Filter to downsample the point cloud data, and Euclidean Cluster Extraction is used to determine the type of the object. The author reported that the model detected the door with 90% precision, and took 0.41 seconds. Yimin et al. [21] developed a door-detection algorithm for an indoor mobile robot pioneer using Kinect sensor data. Here, the author used histogram-based edge detection to identify the door and used bilateral filtering to remove the depth image noise. Furthermore, the author used waypoints to indicate the route of the robot. Fernández-Caramés developed a real-time door-detection system for domestic robot navigation where the author uses haar-like features and integral images to detect the door. The experiment was tested with MoRLaCo mobile robot and detected a door with 4% false positives. Furthermore, the author reports that the false detection ration varies according to distance between the door and the robot [22].

Deep Learning is an emerging technique. It has been widely used for image classification and object detection. Generally, DL has different definition. In zhang et.al [23] clearly described definition of DL. The authors stated that DL is a class of machine learning algorithms that learns the structure between inputs and outputs, besides learning the relationship between two or multiple variables but also the knowledge that manages the relationship as well as the insights that makes sense of the relation. Recently, DL based object detection algorithms are widely used for various automation applications. To pick and place the objects [24], monitoring construction sites [25], recognized trash for cleaning robots [9,26], etc. These were also used for door and door-handle detection.

In [27], Benjamin and Zhang proposed the door-handle detection method for the Stanford AI Robot (STAIR), where the AdaBoost technique and direct feature selection-based cascaded approach was used for door detection. Alejandra et al. used a machine-learning algorithm and contour detection algorithm with spatial filtering technique to detect the door. The experiment was developed for the TurtleBot indoor robot and the detection algorithm obtained 78.0% detection accuracy with a miss-classification rate of 21.93%. Wei et al. [28] proposed a door and door poses detection method for the SIAT robot using a deep-learning framework. The CNN model was trained with 20,500 test images and its detection error was 2.82%. Elen et al. used computer vision and machine-learning technique to identify the door handle and its types where the author used a 2D sliding-window object-detection algorithm and k-means clustering technique for detecting and localizing a door. The experiment was tested with the Star robot, and the model obtained 94.5% recognition and 93.2% localization accuracy, respectively. In another case study, a door and cabinet recognition system was proposed by Maurin et al. for a mobile robot. The author used a 7 ×7 darknet deep-learning model to recognize the door and k-means color clustering for segmentation of the handle point [29].

Another important aspect of the indoor cleaning environment is motion-planning and arm manipulation. Once the vision module can detect and localize the cleaning subject (doors, walls etc.), the information should be exploited by the robot platform such that it will move to the desired location and perform the cleaning task. The authors of [30] proposed a motion-planning strategy for the opening of doors, using the concept of action primitives. In a similar line of work, a motion-planning and arm-manipulation technique was proposed for grasping books from any table, and returning them to a predetermined position [31]. The motion-planning and manipulation for an HSR is discussed in [32,33].

However, the above-mentioned ML and DL studies were focused on door and door-handle detection for a mobile service robot in different applications that were not related to cleaning services. Moreover, very few studies have discussed integrating the vision module data for motion-planning and manipulation. The objective of this case study is to develop a fully autonomous robotic cleaner to conduct cleaning and wiping of door handles to avoid direct contact with germs or viruses, especially in case of virus outbreak. To implement this case study, a lightweight deep-learning framework is developed for a human support robot to detect a door handle. The detection module’s output is exploited by the motion-planning and arm-manipulation algorithms to complete the cleaning task. The experiment was tested in Toyota HSR and its arm part is customized to accomplish the cleaning task. Using a cleaning tool, the robot can spray the disinfecting liquids then clean the sprayed region with a cleaning brush to prevent infections. Furthermore, the indoor path-planning scheme was adopted for a robot to navigate indoors and clean multiple doors. The arm-manipulation function was added to control both the arm and cleaning tool to successfully accomplish the cleaning task.

The paper is organized as follows. Section 2 reviews the proposed system and explains the deep-learning methods used to recognize door handles. This section also describes the cleaning strategy and the arm manipulation. Section 3 presents the validated results in a real scenario. Section 4 presents conclusions and describes future work for this project.

## 2. Proposed System

**Figure 1:**
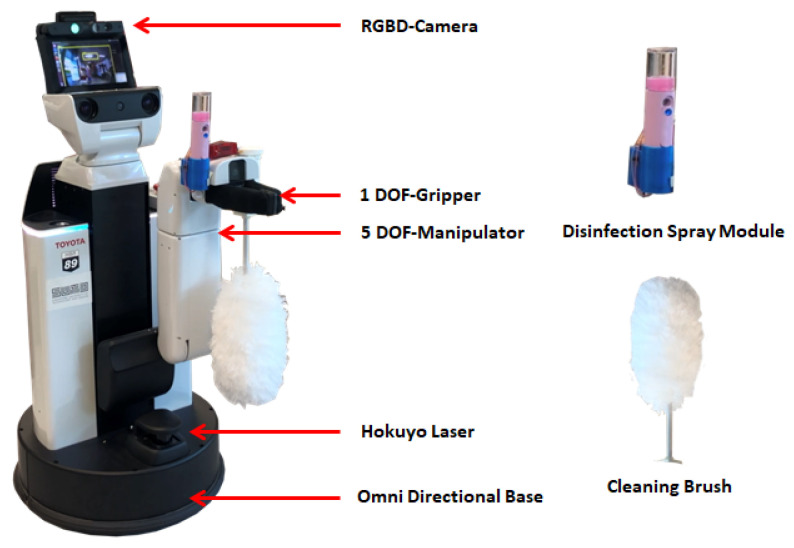
HSR platform with cleaning module.

**Figure 2:**
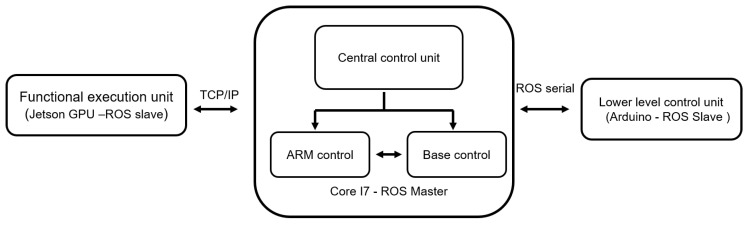
System architecture.

Figure 1 shows the Human Support Robot (HSR) with the customized cleaning module. The system architecture for implementing the door-handle cleaning function in the HSR platform is shown in Figure 2. The details of the HSR platform, functionality of the system architecture and the integration of the functional module in the HSR platform are described as follows.

### 2.1. Overview of HSR

The Human Support Robot (HSR) described in this paper is a product from Toyota Ltd. Co. There is a wide range of sensors installed on the HSR platform. In our work, the HSR ASUS Xtion RGBD camera with RGB and depth perception is used to capture the images in front of the robot. The camera has 1280×1024 resolution and run on 30 frames per second. In addition, the wide-angle camera installed on the manipulator is used to help locate the position of the door handle. IMU and Hokuyo URG-04LX 2D LiDAR are provided in the HSR base for localization and mapping. The HSR is also provided with pneumatic bumpers to prevent any possible collision.

### 2.2. System Architecture

The HSR system architecture block (Figure 2) comprises three computing units—a central control unit, functional execution unit, and lower-level control unit. The ROS API is used in all the layers, which offers the infrastructure and control mechanism that helps hardware components to work easily together. The HSR computing unit comprises of Intel Core i7 CPU (16GB RAM, 256GB SSD) and NVIDIA Jetson TK1 embedded GPU board. The primary control unit uses Linux (Ubuntu) OS and contains the ROS master to control and plan the motion of the robot arms. Furthermore, local connection or data networks are used to establish the communication between ROS nodes and transmit the ROS topic into the ROS network. The ROS transformation frame service function is used to control the vision system of the robot. The functional execution unit carries out the door-handle detection task. NVIDIA Jetson GPU is used in the functional layer to execute the task, connected as a ROS slave and uses TCP/IP to share all the ROS topics.

The last layer is the lower-level controller which executes the cleaning task. It is made from the Arduino control system and is connected as the ROS slave. The layer uses the ROS serial communication package to share all the ROS topics.

### 2.3. Door-Handle Detection

The execution flow of the door handle detection and cleaning process is described in Algorithm 1. In this work, the head RGBD sensor output is used for door-handle detection tasks. Initially, the known objects and their bounding boxes are detected using a customized CNN network trained using RGB images. After detecting the images, the corresponding bounding boxes are used to collect the matching depth images and to project the pixels in 3D space. A point cloud is formed using the projected depth pixels. A ROS object-recognition package is used to remove the pixels belonging to the ground surface or other objects. After removing the points in the detection plane, the point closest to the robot is further moved. By the end, the center point of the current point cloud is assigned as the position of object.
**Algorithm 1:** Algorithm for door handle detection and cleaning  1: **Input:**  2: Image capture from RGBD module  3: **Output:**  4: enable signal to cleaning module  5: **Initialize:**  6: *F* is an Image frame  7: detection_result: is an variable hold the object detection results  8: D: is an variable  9: **Initialize End**  10: **Begin:**  11: **while** (1) **do**  12: **search:**  13: *F* = capture Image (C)  14: detection_result= CNN_Object_ detection(*F*)  15: **if** (detection_result == doorhandle|door) **then**  16:  D=distance_function(door_robot base) ; compute the distance between door and robot base  17:  Move_base(MoveIt(D)) ; move towards door using distance data  18:  goto alignment_check  19: **else**  20:  goto: search  21: **end if**  22: alignment_check:  23: **if** (detection_result == doorhandle) **then**  24:  arm_manipulation (MoveIt) ; enable the arm manipulation to initiate the door handle cleaning  25:  goto: cleaning _enable  26: **else**  27:  base_align( base_movement ) ; adjust the robot base for searching the door handle if door is detect on first frame  28:  *F* = capture Image (C)  29:  *D* = CNN_Object_ detection(*F*)  30:  goto: alignment_check  31: **end if**  32: cleaning_enable:  33: **if** (ARM_synchronize && door_handle_detected) **then**  34:  enable_cleaning module (1) ; enable the cleaning module if ARM position is synchronize with door handle  35:  wait (cleaning_ done)  36: **else**  37:  ARM_synchronize( enable_arm manipulation(MoveIt)) ; enable the arm manipulation function for synchronize arm position with door handle  38:  *F* = capture Image (C)  39:  goto: $cleaning_{e}nable$  40: **end if**  41: **end while**

#### Deep-Learning Framework

A deep-learning-based object-detection framework is used in the HSR platform to recognize the door handle. The framework proposed in this paper is built to essentially identify and classify types of door handles. The base framework used in this paper is YOLO V3 built using Dark Flow in Python. The key advantage is that unlike other object-detection algorithms such as R-CNN, Faster R-CNN, or SSD mobile net, YOLO splits the images into grids. These grids are then visited to spot any objects that resemble the object of interest and a probability value is issued for each grid cell. If the probability value exceeds a specified threshold, then the algorithm detects that as a region containing the desired object. This grid cell is further separated to localize the desired object within that specific grid cell, draw a bounding box, and classify the object. In many other alternative algorithms, the entire image is searched to localize and spot the desired object. This makes the performance of the YOLO much better than its counterparts regarding swiftness and accuracy.

The object-detection network is trained on the training data and its performance is checked using the validation data. In this paper, we have used the loss function as a performance check for the model. If the performance is good, i.e., if the stopping criteria is attained, the trained model would be saved. If not, the training is resumed until the stopping criteria is met. The final step represents implementing the trained model for the real-time scenario in the robot. The trained model is used to process real-time video from the robot’s camera to detect and classify door handles.

**Figure 3:**
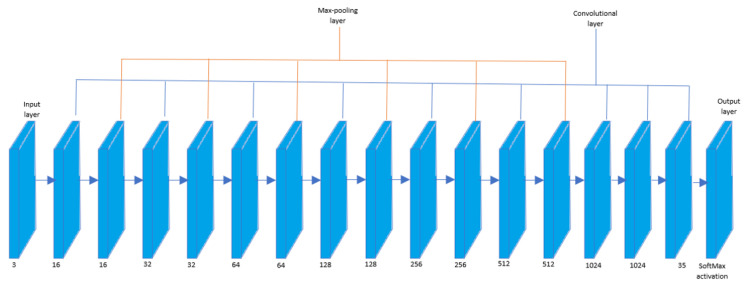
CNN architecture.

Figure 3 shows the architecture for the proposed network. It consists of 10 convolutional layers and 6 pooling layers. It is a general rule of thumb to have a pooling layer after a convolutional layer and therefore the convolutional and pooling layers are placed alternately. The reason for this is to enhance the feature extraction process and to downsample to the spatial dimensions of the image matrix.

### 2.4. Convolutional Layers

Convolution is a widely used method in signal processing, image processing, and other engineering application [34,35]. As the name suggests, this operation convolutes two different functions and calculates the amount of overlap between the functions. In our case, this operation is used to calculate the overlap between a pre-defined filter and the input data. In continuous-time signals, the process of convolution is explained as an integration of two functions that are multiplied together after one of the functions is reversed and shifted by a factor of -τ. Convolutional layers are used to extract higher-level features from images, which would be useful in performing complex object detection functions [36]. This is achieved by systematically applying the user-defined filter throughout an entire image matrix. This lets the filter to detect and extract any features that might be useful in training the model. This is done by performing the convolution operation on each subset of image matrix using the user-defined filter. Equation (1), indicating the convolution operation, is shown below.

(1) $$\left(f*g\right)\left(t\right)=\int_{-\infty }^{\infty }f\left(\tau \right)\;g\left(t-\tau \right)d\tau$$

where *f* and *g* are two variables that are involved in convolution. *f* is the input and *g* is the filter function. Equation (2) explains the convolution operation for discrete time signals [37].

(2) $$x\left(n\right)*h\left(n\right)=\sum_{k=-\infty }^{\infty }x\left(k\right)h\left(n-k\right)$$

where *x* and *h* are two discrete time signals involved in convolution. In discrete-time signals, the convolutions are explained as the sum of element-wise multiplication of two signals when one is reversed and shifted by a factor of −k. The factor of *k* can be customized in the convolution, and this factor is called as stride length. The filter is basically to detect edges in the images which can be extracted as features while updating the weights. The trained model containing the filter with updated weights is used in the testing process, which would detect similar features in the testing images and assist with the classification.

### 2.5. Pooling Layer

Pooling layers are used to reduce the spatial dimension of the input image. More importantly, the pooling layer applied non-linear downsampling and thereby avoided the overfitting of the trained model. In this paper, we have used the max-pooling layer in which the maximum value in the image subset that is overlapped by the filter is taken as the output.

### 2.6. Object Localization

In this paper, we would be using the trained model to detect the door handles in the image. The location of the bounding box is defined by four elements they are interpreted as shown in in (3).

The bounding box representation for each image is stored in an .XML file, and when these labels are inputted into the network for training, they are interpreted as shown in Equation (3).

(3) $$y=\left[\begin{array}{c} P\\ X_{min}\\ Y_{min}\\ X_{max}\\ Y_{max}\\ C_{1}\\ C_{2} \end{array}\right]$$

where *P* is a binary value which determines if there is an object of interest in the image, $X_{min}$ is the upper-left x bounding box coordinate, $Y_{min}$ is the upper-left Y bounding box coordinate, $X_{max}$ is the lower-right x bounding box coordinate, $Y_{max}$ is the lower-right y bounding box coordinate, $C_{1}$ is 1 if the object belongs to class 1, else zero, $C_{2}$ is if the object belongs to class 2, else zero.

The detected bounding box coordinate of the door handle is converted into the 3D word coordinate, which is essential for the robot to manipulate the arm. The bounding box coordinate, i.e., top-right, top-left, bottom-left, and center are the 3D coordinates in the word frame in meters from the base frame of the robot. Here, the projective geometry function is adopted to carry out the image plane to 3D point in the real world coordinate [38]. The translation matrix, such as $T=[t_{1}\;t_{2}\;t_{3}]$ is presumed from the vision module after performing the calibration from the source of world coordinates and the orientation matrix R=[roll,pitch,yawn]. The intrinsic parameter of the camera lies in the focal length $f_{x},f_{y}$, distortion coefficient parameter σ, principle point $\left(x_{c},y_{c}\right)$, and size of pixel (sx,sy); any pixel on the image plane $p=[p_{x}\;p_{y}]$ with 3D coordinate W=[XYd] world plane and W can be calculated by p=H∗W, where H=K∗[RT] is the homogeneous matrix. After predicting the point in the 3D world coordinates frame and setting it aside to the distinct object frame, the ROS transformation frame service package will manage the respective location between this point to the HSR coordinate frame.

### 2.7. Motion-Planning and Arm Manipulation

As the focus of the paper is detection and classification, motion-planning is described for the sake of completeness. Motion/path-planning for a cleaning task can be divided to two decoupled motions of base platform and manipulator. To decouple the motion spaces, the orientation of the manipulator is kept constant while the base platform motion and base platform position is kept constant while performing the cleaning task. Numerous path-planning and arm-manipulation approaches are available in the literature. A technique similar to [30] is proposed for motion-planning and arm manipulation. The notations and symbols are similar to [33]. For that purpose, we represent the base platform pose as ($x_{b},y_{b},\psi_{b}$). The arm height with respect to the base is represented by $z_{arm}$. The roll, pitch, and yaw of the end-effector/gripper is defined as ($\psi_{g},\theta_{g},\phi_{g}$). The orientation of the link connecting the base and gripper is denoted as $\left(\theta_{1},\theta_{2}\right)$. It should be noted that the arm cannot roll independently along the horizontal direction, so $\psi_{g}=\psi_{b}$. The cleaning task is done by manipulating the gripper pitch $\theta_{g}$ and gripper yaw $\phi_{g}$.

### 2.8. Platform Movement

The middle position of the boundary box can be decided from the boundary box data, defined as

$$x_{mid}=\frac{X_{max}+X_{min}}{2},$$

$$Y_{mid}=\frac{Y_{max}+Y_{min}}{2}.$$

The goal of the robot platform is to reach a small neighborhood around the detected door handle mid-position ($X_{mid},Y_{mid}$). The control objective is to decide the desired velocities for the robot platform. By keeping the arms at rest, the platform motion can be moved by a differential drive methodology, i.e.,

(4) $$\dot{p}=\left[\begin{array}{c} {\dot{x}}_{b}\\ {\dot{y}}_{b}\\ {\dot{\psi }}_{b} \end{array}\right]=\left[\begin{array}{cc} cos(\psi_{b}) & 0\\ sin(\psi_{b}) & 0\\ 0 & 1 \end{array}\right]\left[\begin{array}{c} v_{b}\\ \omega_{b} \end{array}\right]$$

The robot can reach near ($X_{mid},Y_{mid}$) by setting the desired positions as ($X_{mid}\pm \epsilon ,Y_{mid}\pm \epsilon$), where ϵ is a positive number to be tuned according to arm length and interior geometry. The desired velocities ($v_{b},\omega_{b}$) are the output of a dynamic feedback linearizing controller. The input velocity is decided through the integrator defined as:

(5) $$\dot{\zeta }=f,\;\;v_{b}=\zeta .$$

The forward kinematics can be rewritten as:

(6) $$\left[\begin{array}{c} {\ddot{x}}_{b}\\ {\ddot{y}}_{b} \end{array}\right]=\left[\begin{array}{cc} cos(\psi_{b}) & -v_{b}sin\left(\psi_{b}\right)\\ sin(\psi_{b}) & v_{b}cos\left(\psi_{b}\right) \end{array}\right]\left[\begin{array}{c} f\\ \omega \end{array}\right]$$

The overall control law can be decided as:

(7) $$\begin{array}{l} \dot{\zeta }=f,\;\;v_{b}=\zeta \\ f={\ddot{X}}_{mid}-k_{1}\frac{d(x_{b}-X_{mid})}{dt}-k_{2}\left(x_{b}-X_{mid}\pm \epsilon \right)\\ \omega ={\ddot{Y}}_{mid}-k_{1}\frac{d(y_{b}-Y_{mid})}{dt}-k_{2}\left(y_{b}-Y_{mid}\pm \epsilon \right) \end{array}$$

where $k_{1},k_{2}\in R^{+}$. With the control law (7), the closed-loop system can be described as:

(8) $$\begin{array}{l} {\ddot{x}}_{b}-{\ddot{X}}_{mid}=-k_{1}\frac{d(x_{b}-X_{mid})}{dt}-k_{2}\left(x_{b}-X_{mid}\pm \epsilon \right)\\ {\ddot{y}}_{b}-{\ddot{Y}}_{mid}=-k_{1}\frac{d(y_{b}-Y_{mid})}{dt}-k_{2}\left(y_{b}-Y_{mid}\pm \epsilon \right) \end{array}$$

The differential Equation (8) describes a stable error dynamic, and hence the robot platform reaches an ϵ neighborhood of the door handle, i.e.,:

$$x_{b}\to X_{mid}\pm \epsilon ,\;y_{b}\to Y_{mid}\pm \epsilon .$$

### 2.9. Arm Manipulation

The motion-planning for the manipulator should be programmed by keeping in mind the workspace of the arm. Hence, before the cleaning task starts, the base platform should reach the ϵ neighborhood of the door handle. This can be evaluated by using the arm camera on the HSR. If the robot has reached the door handle, the arm camera can properly capture its image. Once this step is verified, the cleaning task can be done. There can be various shapes of the door handles, but the cleaning is carried out using data from the bounding box. The arm manipulation for the cleaning is done using the following two steps.

(1) Define the door-handle position as $\left(x_{mid},y_{mid},z_{mid}\right)$. Once the arm camera detects the handle, this means the $\left|\right|x_{b}-x_{mid}\left|\right|\leq \epsilon$ and $\left|\right|y_{b}-y_{mid}\left|\right|\leq \epsilon$. The error between the door-handle position and arm gripper’s position can be given by

$$\left(\epsilon ,\epsilon ,d_{z}\right)$$

where $d_{z}$ represents the error between the height of the gripper and $z_{mid}$. These errors can be transformed into joint space by

(9) $$\left[\begin{array}{c} d\theta_{1}\\ d\theta_{2}\\ d\theta_{g}\\ d\psi_{g}\\ d\phi_{g}\\ d\psi_{b} \end{array}\right]=J^{-1}\left[\begin{array}{c} \epsilon \\ \epsilon \\ d_{z}\\ 0\\ 0\\ 0 \end{array}\right]$$

where *J* is the Jacobean of the robot arm. The error in joint space is fed back through a PID to actuate the robot arm. After this stage, the arm camera should be able to detect the door handle, and the image of the door handle should be on the center pixels of the image matrix. This step should be repeated until a desirable distance between the door handle and arm gripper is reached.

(2) After Step 1, one should get

$$x_{b}\approx x_{d},\;y_{b}\approx y_{d},\;z_{arm}\approx z_{d}$$

where $x_{d},y_{d},z_{d}$ are desired distances from the door. The cleaning should be performed only by actuating the gripper, without moving the base. The following actions should be repeated a fixed number of times depending on cleaning requirements.

(I) At this position, give an error signal $\left(0,0,z_{mid}+l_{h}/2\right)$ ($l_{h}$ is the approximate length of the door handle), and solve the inverse kinematics (9) to transform this error into joint space. Feed the error into a PID controller, and exploit the output to actuate the gripper.

(II) Give an error signal $\left(0,0,z_{mid}-l_{h}/2\right)$, and follow Step 2 (I).

### 2.10. Navigation

A two-dimensional map of the environment is generated by ROS hector-slam using the data from 2D LiDAR and IMU sensors from HSR. To localize itself at every time-step in the environment, an Adaptive Monte Carlo Localization (AMCL) algorithm is used. Both a static and dynamic obstacle map is needed to generate a combined obstacle map (costmap) for the proper navigation of HSR in the environment. A static obstacle map is the 2D map already generated using hector-slam. LIDAR and RGBD sensors update the dynamic obstacle map. Once the target is fixed, the path is generated using the move_base algorithm on the combined obstacle map. As the robot has a global path to follow and a costmap, the local planar generates the command velocities, (vx, vy and vθ) based on the global plan and costmaps, to navigate the HSR to the target position along the planned path.The following ROS packages are used to accomplish the navigation tasks, amcl, base_local_planner, base_local_planner, clear_costmap_recovery, costmap_2d, dwa_local_planner, fake_localization, global_planner, map_server, move_base, move_base_msgs, move_slow_and_clear, navfn, nav_core, robot_pose_ekf, rotate_recovery, voxel_grid.

### 2.11. Cleaning Module

**Figure 4:**
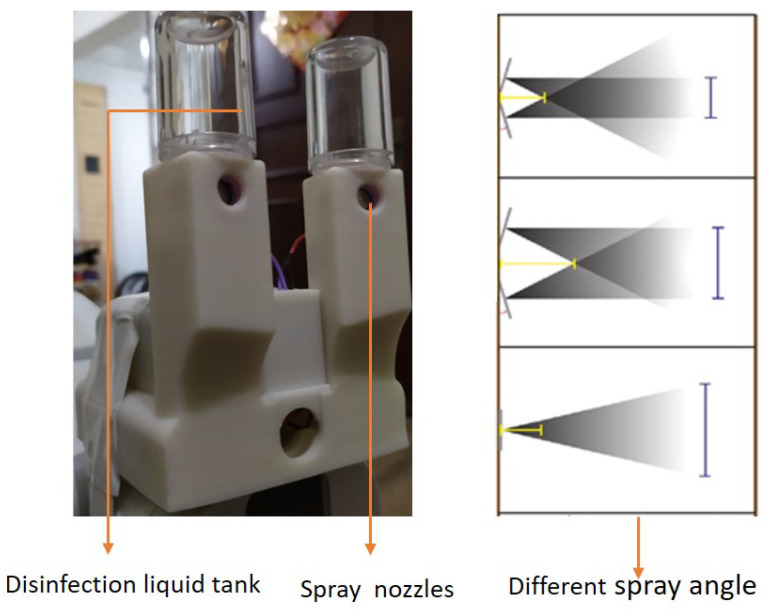
Disinfectant liquid spraying unit.

The cleaning module comprises a disinfectant liquid spraying unit and a spindle-type cleaning brush; both units were attached to the HSR manipulator as shown in Figure 1. The spraying unit is constructed with two spraying guns and a separate disinfection liquid tank. To spray the disinfectant, the disinfectant was drained by a small sucking unit from a tank and forwarded to an electric resistance boiler circuit to convert into steam. Then, the generated steam is sprayed onto the door handle through two spray-gun nozzles (Figure 4) at different angles. After spraying the disinfectant liquid on the door handle, the cleaning brush is enabled for wiping the sprayed area. A horizontal and vertical zigzag cleaning pattern is adopted to spray the disinfectant and wipe function. To wipe the entire sprayed region, the cleaning brush was rotated 360 degrees through a 12-volt DC motor. The function of the spraying unit and cleaning brush is controlled by an Arduino Mega micro-controller and HSR task-scheduler module. Here, the task scheduler selects the module to be enabled to perform the disinfection task and the Arduino Mega micro-controller executes the task by generating the relay control signal for the spraying unit and PWM generation signals for enabling the DC motor. The tanks can be easily removed for maintenance and changing the cleaning substance, as required by the cleaning cycle.

## 3. Experimental Results

**Figure 5:**
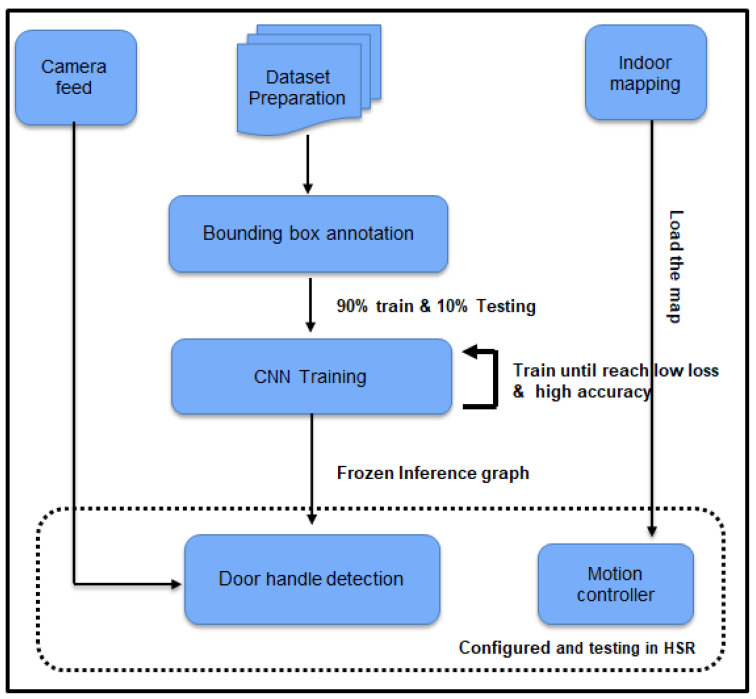
Block diagram of experimental procedures.

This section describes the experimental results of door detection and the cleaning mechanism. Figure 5 shows the block diagram of of experimental procedure. The experiment has three phases, which are training the deep-learning framework, mapping the indoor environment, and configuring and configuring and testing with HSR robot platform.

Here, the dataset preparation process involves collecting the different door images at high resolution. The dataset contains about 4500 images collected online through Bing image search, the Miguel et al. [39] door dataset and also our institution’s different door handles. The data set images are collected in the robot perspective with Intel real-sense depth camera at different angles and different lighting conditions. Furthermore, the collected door datasets are sorted into three classes, such as circle type, lever type, and bar type handles. To balance the images, 1200 images are used for each class in the training, and 300 images are used for validation. Image resolution of 640×480 was used in the experiments for both training and testing the CNN model. Furthermore, data expansion techniques are used to enhance the CNN learning rate and controlling overfitting on training phase. In the data enhancement process, rotation, scaling, and flipping are applied on the collected image. K-fold (here K = 10) cross-validation process is involved for assessing the model. In this assessment process, the dataset is divided into 10 sections; 9 of these sets are used for training the model, and the remaining one is used for testing the model. This process is repeated 10 times to eliminate any biasing conditions due to particular training or testing datasets. The results from the performance metrics are repeated 10 times and the means of the results are provided. The resulting images from the highest-accuracy models are provided here. The deep-learning models were developed in Tensor-flow 1.9 Ubuntu 18.04 version and trained using the following hardware configuration: Intel core i7-8700k, 64 GB RAM, and Nvidia GeForce GTX 1080 Ti Graphics Cards.

Standard statistical measures are used to assess the performance of the proposed scheme. These include accuracy, precision, recall, and $F_{measure}$, which are computed using the equations below:

(10) $$Accuracy\left(Acc\right)=\frac{tp+tn}{tp+fp+tn+fn}$$

(11) $$Precision\left(Prec\right)=\frac{tp}{tp+fp}$$

(12) $$Recall\left(Rec\right)=\frac{tp}{tp+fn}$$

(13) $$F_{measure}\left(F_{1}\right)=\frac{2\times precision\times recall}{precision+recall}$$

Here, tp,fp,tn,fn represent the true positives, false positives, true negatives, and false negatives, respectively, as per the standard confusion matrix.

### 3.1. Validation of Door-Detection Model

**Figure 6:**
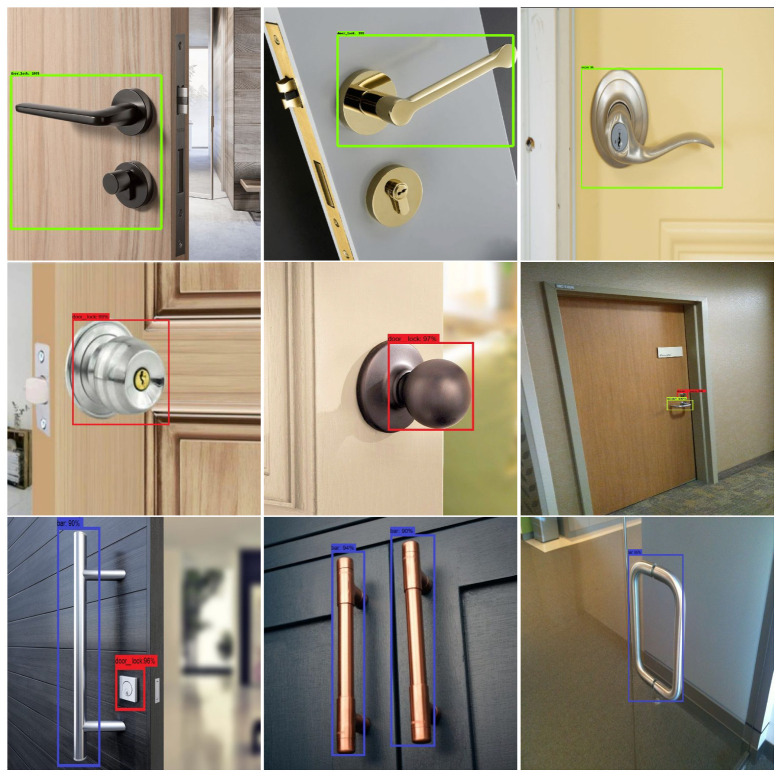
Offline test results.

After successfully training the model, the detection accuracy of the trained model was tested offline and in real time with the HSR robot. To perform the offline test, the model inference graph was configured into the HSR GPU and tested with 120 test images. The detection results of the offline tested images are shown in Figure 6. Here, lever-type door-handle detection is marked by a green rectangle box, circle-type door-handle detection is marked by a red rectangle box, and bar type is marked by a blue rectangle box. In this analysis, the trained CNN model detected most of door handles accurately with above 95% higher confidence level.

**Figure 7:**
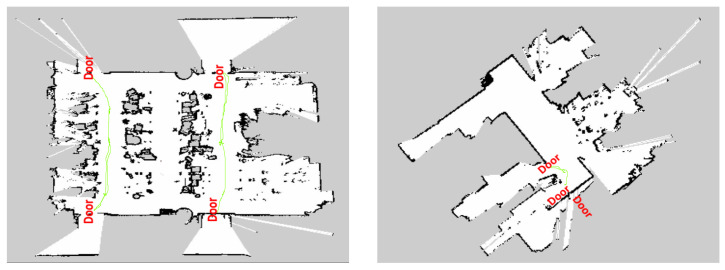
Localization result for test bed1 and test bed2.

**Figure 8:**
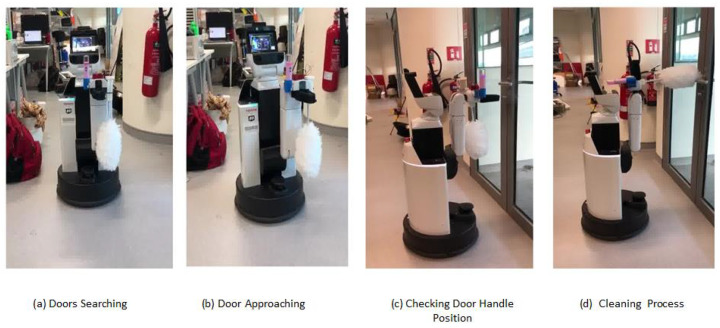
Real-time door-handle cleaning demonstration.

For real-time door-handle disinfection trail, two indoor environments were chosen as test bed; those environments were first mapped manually by mapping algorithm, with HSR and door location information stored as wave-point data. Our first test bed has four doors fixed with a lever-type door handle and another test bed has three doors, all fixed with a circle-type door handle, the localization result was shown in Figure 7. Figure 8 shows the real-time door-handle cleaning task (test bed1) accomplished by HSR, where Figure 8a,b shows the HSR navigation and door-handle detection and Figure 8c,d demonstrates the spraying of disinfection liquid and cleaning of the door handle.

**Figure 9:**
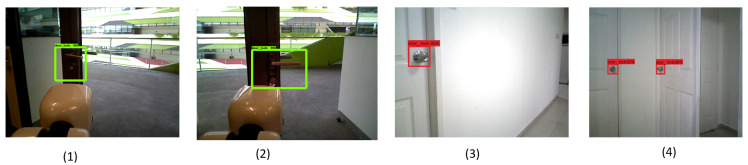
Real-time door-handle detection—(1, 2 Test bed1), (3, 4 Test bed2).

In this trial, the HSR RGBD vision system was used to capture the door runs at 10 fps, and image resolution was set to 640×480. The robot was operated in autonomous mode and door-handle detection and navigation was recorded from remote console. The experiment was tested with different lighting conditions and its detection results are shown in Figure 9. The experiment was trailed with different lighting conditions and it was observed that the detection model detects door handles with a confidence level of 88 to 92%.

The statistical measures indicate that the trained CNN model has detected the door handle with an average of 94.56% accuracy for the offline test and 91.2% average accuracy for the online test. The study ensures that the proposed system was not heavily affected by environmental factors such as varying lighting conditions, but slightly affected by the mirror effect. However, the mirror issue has been resolved by fusing depth information with the detection results.

Furthermore, statistical measures have been performed for estimating the robustness of the detection model for both the online and offline experiments. Table 1 shows the statistical measure results for online and offline experiments.

### 3.2. Execution Time

Table 2 show the processing time of the detection model and cleaning execution time of the HSR robot. The cleaning execution time was computed based on the time taken by the HSR to perform a disinfection task on test bed 1 doors and test bed 2 doors. Here, the workspace configuration time, object-detection time, and cleaning time is considered to estimate the cleaning-time computation.

The table results indicate that the proposed CNN framework took an average of 11.07 s to detect the door handle in 180 test images, and took 18 and 10 min to clean the test bed 1 and test bed 2 door handles, respectively.

### 3.3. Comparison with Other Object Detection Framework and Existing Schemes

Table 3 shows the comparative analysis of the proposed object detection framework with other object detection frameworks includes SSD Mobile Net, SSD Inception. The detection and classification accuracy of two networks has been trained using the same data-set for a constant amount of time. Standard metrics were used for the performance estimation of these methods, as shown in Table 3.

SSD-MobilNet and proposed object detection framework had almost the same accuracy. However, the execution time and detection accuracy of SSD-Inception is higher than the proposed framework. Our proposed system has achieved better trade-off between accuracy and computational cost with reduced execution time.

To the best of our knowledge, there is no similar work for comparing our proposed system performance metrics. Hence we consider the similar door handle detection schemes for comparative analysis. In this contest, we took a learning-based door handle scheme for comparison analysis, and models’ detection accuracy was considered for evaluation metrics. The detection result of various door handle detection schemes is shown in Table 4.

From the above table it is evident that our model has shown better performance metrics compared to other models. In our work, the darkflow framework was used to build the CNN model. To improve the detection accuracy, we fixed the low learning rate of existing works and also used a reduced number of hidden layers after the convolution and pooling layer.

### 3.4. Reliability, Robustness, and Resilience Analysis

The critical aspects of a system that determine the overall performance are reliability, robustness, and resilience [43]. The robustness of the proposed method might be affected due to occultation. This shortcoming could be eliminated by designating different approaching direction for a single door. For example, if the door handle is not detected from a defined designated position, the robot could move to the next location to detect the door. The continuous navigation of the robot for a long time may increase the risk of the localization error. In the proposed method, the navigation functionality of the robot is used only for navigating near a door. The vision system could detect a door when the robot moves near to the door. After detecting the door, the position information of the door is perceived as a 3D coordinates through the vision system. Then the robot manipulation and navigation required for cleaning based on the perceived 3D coordinates. Therefore, the navigation error would not affect the reliability of the door cleaning process. Navigation toward a door on an already defined navigation path would be failed due to a blockage caused by an unmapped obstacle. In such a situation, HSR can detect the unmapped obstacle through lidar information to avoid the collision and subsequently reroute the navigation toward the door. This behavior ascertains the resilience of the robot in case of unmapped obstacles. As future work, we plan to conduct a detailed analysis of the proposed system in terms of reliability, robustness, and resilience, and ways to improve the performance in those aspects.

## 4. Conclusions

This work proposed a novel technique for door-handle cleaning automation using the Toyota HSR platform. The detection and classification algorithm used a CNN based deep learning methodology, which generates a set of coordinates surrounding the door handles. These coordinates are exploited, to generate the operational space of the robot using ROS, and to develop manipulator motion planning. The veracity of the technique is tested offline and online through a series of simulations and experiments. The accuracy of the detection is calculated to be more than 90% in both cases. The real-time experiments are performed on two different testbeds successfully, which confirmed the efficiency of the proposed framework. It can be concluded that the proposed system has the potential for cleaning door handles in the indoor and public places, which in turn helps to overcome the shortage of manpower in the cleaning industry. Future aspects of this work involve obstacle avoidance, human interaction and safe navigation.

## Figures and Tables

**Table 1:** Statistical measures for door-handle detection.

Test	Lever Type Handle				Circle Type				Bar Type Handle			
	Prec.	Recall	$F_{1}$	Accuracy	Prec.	Recall	$F_{1}$	Accuracy	Prec.	Recall	$F_{1}$	Accuracy
offline	97.2	95.8	95.1	95.5	95.5	93.8	93.5	94.3	96.5	94.4	93.9	93.9
Real time	91.2	90.6	89.7	90.4	92.9	91.7	91.3	92.0	NA	NA	NA	NA

**Table 2:** Execution time analysis.

Task	Execution Time
Inference time (offline—180 images)	11.07 (s)
Test bed1 (4 doors)	18 (min)
Test bed2 (3doors)	10 (min)

**Table 3:** Comparison with other object detection framework.

Object Detection Framework	Precision	Recall	F1	Accuracy	Computation Time (s)
SSD MobileNet	96.05	95.80	95.49	95.22	15.88
SSD Inception	97.55	97.13	97.07	97.00	26.03
Proposed (180 images)	97.20	95.8	95.1	95.5	11.07

**Table 4:** Comparison with existing door-handle detection schemes.

Case Study	Application	Algorithm	Detection Accuracy
Jauregi [40]	Tartlo robot, door open task	circle hough transform + Oblique Classifier-1	85
Liang et al. [41]	Visually Impaired	Yolo V2	80.00
Maurin et al. [29]	door-handle open task, iRobot-ATR V-Jr	7 × 7 × 12 multi-layer CNN + k-means clustering	92.00
Ellen et al. [42]	Door open case study (stair robot)	2d-sliding window	93.20
Proposed system	door handle cleaning	16-layer CNN	94.56
